# Mr. Henry O'Halloran Giles

**Published:** 1911-04-22

**Authors:** 


News has arrived at Melbourne of the death of Mr. Henry O'Halloran Giles, late of Glenelg, South Australia, at the age of forty-two. Mr. Giles took his diploma at London in 1894, having come from Australia, where he had gained the degree of M.B., Ch.B. of Adelaide University.

